# The Frequency of Intraocular Pressure Elevation, Incidence of Secondary Glaucoma, and Surgical Treatment With Postoperative Complications in Pediatric and Adult Patients With Uveitis

**DOI:** 10.7759/cureus.55734

**Published:** 2024-03-07

**Authors:** Cem Evereklioglu, Tülay Karacan Erşekerci, Hatice Kübra Sönmez, Hidayet Sener, Duygu Gulmez Sevim, Osman Ahmet Polat, Hatice Arda, Fatih Horozoglu

**Affiliations:** 1 Department of Ophthalmology, Division of Uvea-Behçet Unit, Medical Faculty, Erciyes University, Kayseri, TUR; 2 Department of Ophthalmology, Kayseri Training and Research Hospital, Kayseri, TUR

**Keywords:** anti-metabolites, filtration surgery, behçet disease, inflammatory glaucoma, uveitis

## Abstract

Purpose

To determine the etiology and anatomic localization of uveitis, the frequency of intraocular pressure (IOP) elevation, and the type of secondary glaucoma and to assess the medical, surgical, and postoperative complications in adult and pediatric patients with acute or chronic uveitis.

Methods

A total of 307 eyes of 186 patients who were followed up in the Uvea-Behçet Unit of the Ophthalmology Department, Erciyes University, Turkey, were included in the study. Demographic, ocular, and systemic data were recorded; ophthalmological examinations were performed; and recurrences and complications of uveitis were identified. The eyes with IOP over 22 mmHg, types of secondary glaucoma, their etiologies, efficiency of medical and surgical treatments, and complications were recorded.

Results

The mean age was 33 ± 12 years (range: 6-65). Of the 186 patients, diagnoses were as follows: idiopathic uveitis in 84 (45.2%), Behçet disease in 65 (34.9%), ankylosing spondylitis in eight (4.3%), juvenile idiopathic arthritis in five (2.7%), herpetic keratouveitis in three (1.6%), Fuchs iridocyclitis in three (1.6%), Vogt-Koyanagi-Harada syndrome in three (1.6%), tuberculosis uveitis in three (1.6%), Crohn disease in three (1.6%), ocular toxoplasmosis in two (1.1%), multiple sclerosis in two (1.1%), Lyme disease in two (1.1%), rheumatoid arthritis in two (1.1%) and tubulointerstitial nephritis in one patient (0.5%). Secondary glaucoma was detected in 67 (21.9%) of 307 eyes, which developed in 13.7% and 26.8% of the eyes with acute and chronic uveitis, respectively. Of 67 eyes, it was open-angle glaucoma in 58 (86.5%), angle-closure glaucoma in six (9.0%), and neovascular glaucoma in three (4.5%). Control of IOP was achieved by medical therapy in 53 eyes (79.1%) and by surgery in 12 eyes (17.9%), whereas evisceration was required in two eyes (3.0%). Laser iridotomy was performed in four eyes (33.4%), trabeculectomy with mitomycin-C (MMC) in six eyes (50.0%), laser iridotomy plus trabeculectomy with MMC in one eye (8.3%), and express mini shunt implantation in one eye (8.3%). After surgery, IOP was controlled without anti-glaucomatous agents in six eyes (50%) and with anti-glaucomatous agents in the remaining six eyes (50.0%).

Conclusion

Secondary glaucoma is one of the most important complications of uveitis and may result in severe visual impairment. Early diagnosis and appropriate treatment can prevent these potential complications.

## Introduction

Uveitis is the inflammation of uveal tissue, which has immunological and systemic components with some complications that may progress to complete blindness if untreated on time [1,2]. Secondary glaucoma is one of the most blinding complications of the disease, which is commonly encountered as a result of anterior uveitis and panuveitis with a lesser degree from posterior uveitis and pars planitis [3]. Intraocular pressure (IOP) can be found as low, normal, or high due to the effect of inflammation during uveitis attacks [4]. Ciliary body inflammation reduces IOP by decreasing the production of humor aqueous, whereas polymorphonuclear leukocytes and macrophages recruited to the inflammation site by T cells block the drainage of humor aqueous, resulting in an IOP increase [5]. Similarly, inflammatory mediators such as arachidonic acid metabolites, nitric oxide, cytokines, and free oxygen radicals may play a role during the secondary glaucoma development, which may impair the blood-aqueous barrier with an increased protein concentration in humor aqueous and outflow resistance [6]. Although prolonged corticosteroid usage may also cause transient or persistent IOP increase, morphological changes such as irido-lenticular synechia, pupillary block, and anterior rotation of ciliary body may cause secondary angle-closure glaucoma, resulting in permanent optic disc and nerve changes [7].

The treatment of glaucoma secondary to uveitis is highly difficult, and the goal of the therapy is to prevent permanent structural changes in the optic nerve [6,7]. Therefore, the etiology of uveitis should be determined to plan an appropriate treatment regime, and the uveitis should immediately be treated first [8-10]. In cases of glaucoma, medical therapy is attempted initially, and then surgical modalities should be preferred in unresponsive cases to medical treatment.

The aim of this study is to determine the etiology and anatomic localization of uveitis, frequency of IOP elevation, incidence and type of secondary glaucoma, medical and surgical treatment modalities, and postoperative potential complications in pediatric and adult patients with uveitis in a tertiary eye center.

## Materials and methods

One hundred and eighty-six pediatric or adult patients with the diagnosis of uveitis were managed in the Department of Ophthalmology, Division of Uvea-Behçet Unit, Erciyes University, Turkey. The study conformed to the tenets of the Declaration of Helsinki, and the study was approved by the Institutional Review Board (IRB) of Erciyes University (approval number: 2023/459).

The inclusion criteria were as follows: adult or pediatric patients who underwent surgery due to glaucoma secondary to uveitis, a preoperative follow-up period of a minimum of 12 months, and patients who attended follow-up visits postoperatively. Exclusion criteria were as follows: patients with non-uveitic glaucoma; patients who had a single acute uveitis attack, traumatic uveitis, or lens-related uveitis; and patients who did not attend routine postoperative follow-ups.

A routine thorough medical history was obtained, and all patients underwent a complete ophthalmological examination, comprising best-corrected visual acuity, slit-lamp biomicroscopic examination, IOP measurements, gonioscopy, and dilated fundus and optic nerve head evaluations. The clinical diagnosis of uveitis was made, and its etiology was investigated by anamnesis, related laboratory evaluations, and consultations. Three IOP measurements were performed on different days. The diagnosis of secondary glaucoma was made according to elevated IOP over 22 mmHg in the presence of typical glaucomatous visual field defect by perimetry with glaucomatous optic disc abnormality in Heidelberg retinal tomography. The diagnosis of uveitis was made on the clinical findings of at least one active inflammatory chorioretinal or retinal vascular lesion, anterior chamber cell, or vitreous haze, according to the Standardisation of Uveitis Nomenclature (SUN) Working Group and adapted National Eye Institute criteria under the regimen [7,11,12]. The exclusion criteria were as follows: patients with other retinal diseases that may adversely affect visual acuity other than uveitis and patients with cataract surgery during the last six months. Patients with transient IOP elevation during or after topical, oral, subtenon, and intravitreal corticosteroid therapies for uveitis attacks were not considered secondary glaucoma, and these cases were considered to be transient IOP elevation secondary to inflammation and corticosteroid therapy.

Patients with secondary glaucoma were classified as open-angle glaucoma, secondary angle-closure glaucoma due to pupillary block, and neovascular glaucoma. Medical therapy was initiated in patients with secondary open-angle glaucoma as first-line therapy, whereas laser iridotomy was performed in patients with angle-closure glaucoma with seclusio pupillae. Glaucoma surgery was performed in eyes unresponsive to medical therapy with visual field loss. In addition, patients with glaucomatous optic nerve progression had glaucoma surgery.

Surgical procedure

Trabeculectomy was performed with a fornix- or limbal-based conjunctival flap in the superior quadrant. Subconjunctival application of 0.04% mitomycin-C (MMC) was performed for 2 min, followed by a thorough wash with a balanced salt solution. The scleral flap was closed with a 10.0 non-absorbable suture, whereas the conjunctiva was closed with 8-0 Vicryl interrupted sutures.

Statistics

All statistics in the present study were performed using Statistical Product and Service Solutions (SPSS, version 21.0; IBM Corp., Armonk, NY). Numeric variables were presented as mean±standard deviation or median (25th and 75th percentiles). Normal distribution was assessed by using the Shapiro-Wilk test. In the variables with normal distribution, Student's t-test was used to compare groups. Repeated measurements were compared by using the dependent t-test. The chi-square exact test was used in the comparison of two qualitative variables. A p-value less than 0.05 was considered to be significant.

## Results

A total of 307 eyes of 186 patients (99 male, 87 female) were included in the study. Of 186 patients, uveitis was unilateral in 65 (right=33, left=32) and bilateral in 121 (Table 1). The mean age at presentation was 33±12 years (range: 6-65), and the mean follow-up time was 63±44 months (range: 12-216). The etiology was idiopathic in 85 patients (45.6%), Behçet disease in 65 (34.9%), ankylosing spondylitis in eight (4.3%), juvenile idiopathic arthritis in five (2.7%), herpes keratouveitis in three (1.6%), Fuchs iridocyclitis in three (1.6%), Vogt-Koyanagi-Harada syndrome in three (1.6%), tuberculosis uveitis in three (1.6%), Crohn disease in three (1.6%), ocular toxoplasmosis in two (1.1%), multiple sclerosis in two (1.1%), Lyme disease in two (1.1%), rheumatoid arthritis in two (1.1%), and tubulointerstitial nephritis in one (0.5%). The localization was anterior uveitis in 84 patients (45.2%), intermediate uveitis in 14 (7.5%), posterior uveitis in 18 (9.7%), and panuveitis in 70 (37.6%). The onset of uveitis was acute in 73 patients (39.2%) and chronic in 113 (60.8%) cases.

**Table 1:** Demographic characteristics of patients with uveitis and secondary glaucoma. IOP=Intraocular pressure

Uveitic patients (n=186)	
Gender (male/female)	99/87
Laterality (unilateral/bilateral)	65/121
Onset of uveitis (acute/chronic)	73/113
Mean age of uveitis (years)	33±12 (range: 6-65)
Mean follow-up time (months)	63±44 (range: 12-216)
Median interval between uveitis glaucoma (months)	12 (range: 1.5-36)
Number of patients with secondary glaucoma	42 (67 eyes)
Mean preoperative IOP (mmHg)	34±5 (26-40)
Mean postoperative IOP (mmHg)	13±9 (6-16)

Of the 307 eyes with uveitis, short-term transient IOP elevations (range: 2-24 months) were present in 11 eyes (3.5%) as a result of inflammation and topical and/or systemic corticosteroid usage, whereas this elevation was seen secondary to subtenon or intravitreal corticosteroid injections in seven eyes (2.3%), all of which returned to normal in due course with appropriate anti-glaucomatous agents.

Of 307 eyes of 186 patients, secondary glaucoma was detected in 67 eyes (21.8%) of 42 patients (unilateral in 17, bilateral in 25). The median interval between uveitis and the diagnosis of glaucoma was 12 months (range: 1.5-36). When 67 eyes with secondary glaucoma were evaluated, the etiology was Behçet disease in 31 (46.2%), idiopathic glaucoma in 28 (41.8%), ankylosing spondylitis in 2 (3.0%), Fuchs iridocyclitis in two (3.0%), multiple sclerosis in two (3.0%), and Crohn disease in two (3.0%). The localization of uveitis in these 67 eyes with secondary glaucoma was panuveitis in 38 eyes (56.7%), anterior uveitis in 28 eyes (41.8%), and intermediate uveitis in one eye (1.5%). Of 67 eyes, it was found that there was open-angle glaucoma in 58 (86.5%), angle-closure glaucoma secondary to pupillary block in six (9.0%), and neovascular glaucoma in three (4.5%) (Table 2).

**Table 2:** The etiology, localization, and type of uveitis in eyes with secondary glaucoma.

Etiology of uveitis	n=67 eyes	Percent (%)
Behçet Disease	31	46.2
Idiopathic	28	41.8
Ankylosing spondylitis	2	3.0
Fuchs iridocyclitis	2	3.0
Multiple sclerosis	2	3.0
Crohn disease	2	3.0
Localization of uveitis		
Anterior uveitis	28	41.8
Intermediate uveitis	1	1.5
Posterior uveitis	-	-
Panuveitis	38	56.7
Type of glaucoma		
Open-angle glaucoma	58	86.5
Angle-closure glaucoma	6	9.0
Neovascular glaucoma	3	4.5

Initial treatment was topical anti-glaucomatous agents in all eyes with secondary glaucoma, which was controlled in 53 eyes (79.1%). In the remaining cases, glaucoma surgery was required in 12 eyes (17.9%) in which IOP was not controlled with the progression of visual field defect and optic nerve cupping despite maximal medical treatment. Uveitis attacks were controlled at least three months before surgery. Evisceration was performed in two eyes (3.0%) at the terminal phase. In these 53 eyes without surgery, IOP was controlled by ß-blocker+carbonic anhydrase inhibitor (CAI) combination within 30 eyes (56.7%); by ß-blocker+CAI combination and sympathomimetic in 19 eyes (35.8%); by ß-blocker and sympathomimetic in two eyes (3.7%); by ß-blocker alone in one eye (1.9%); and by topical CAI alone in one eye (1.9%) (Table 3).

**Table 3:** The control of intraocular pressure by medication in eyes with secondary glaucoma without surgery. CAI=carbonic anhydrase inhibitor

Medications	n=53	Percent (%)
β-blocker+CAI combination	30	56.7
β-blocker+CAI combination+sympathomimetic	19	35.8
β-blocker+sympathomimetic	2	3.7
β-blocker	1	1.9
Topical CAI	1	1.9

In the eyes with secondary glaucoma, laser iridotomy was performed in four eyes (33.4%), trabeculectomy with MMC in six eyes (50.0%) including two children, iridectomy followed by trabeculectomy with MMC in one eye (8.3%), and express mini shunt in one eye (8.3%) (Table 4).

**Table 4:** Surgical techniques used in uveitis patients with secondary glaucoma and used medications. CAI=carbonic anhydrase inhibitor, LI=laser iridotomy, MMC=mitomycin-C, TRAB=trabeculectomy

Type of surgical intervention	n=12 eyes	Percent (%)	β-blocker+CAI	β-blocker+CAI+sympathomimetic	No drug
LI	4	33.4	1	3	None
TRAB with MMC	6	50.0	1	None	5
LI+TRAB with MMC	1	8.3	None	1	None
Express mini shunt	1	8.3	None	None	1

Preoperative IOP measured by the Goldmann applanation tonometer varied from 26 to 40 mmHg (mean: 34±5 mmHg). The IOP measured on the first day after surgery varied from 5 to 30 mmHg (mean: 13±9 mmHg), whereas the IOP measured at the last visit varied from 6 to 16 mmHg (mean: 11±3 mmHg). The difference between preoperative and postoperative IOPs was found to be significant (for each, p=0.001). Of the four eyes that underwent laser iridotomy, the IOP was controlled by ß-blocker+CAI combination in one eye, whereas an additional sympathomimetic was required in the remaining three eyes (Table 4). In two patients who had trabeculectomy with MMC, no complication was observed in the postoperative period, and the IOP was controlled in both patients without the need for anti-glaucomatous agents. Similarly, bevacizumab was injected into the anterior chamber in a patient with Behçet disease and neovascular glaucoma who had trabeculectomy with MMC afterward. The IOP was controlled postoperatively without the need for anti-glaucomatous agents. Normal IOP was obtained without the need for anti-glaucomatous agents in a patient who underwent express mini shunt. However, bleb revision was needed in one case due to persistent IOP below 6 mmHg over one month. In this single case, subconjunctival autologous blood was injected under the bleb two times as hypotonia persisted with normal IOP levels in due course.

In another patient's trabeculectomy, bleb revision was performed because IOP remained above 22 mmHg postoperatively, which required a second trabeculectomy with MMC 8 months after the first operation. Although choroidal detachment developed in this case due to IOP below 6 mmHg, it recovered with medical treatment at two months without the need for surgical intervention with normal IOP levels. Two patients had end-stage ocular Behçet disease with frequent attacks, who could hardly be controlled by medications, including cyclosporine, azathioprine, and systemic corticosteroids. The most commonly used method was trabeculectomy with MMC in seven uveitis patients with or without laser iridotomy. Evisceration was required in two patients with absolute painful neovascular glaucoma.

## Discussion

Secondary glaucoma has been reported in patients with uveitis between the rates of 5.2% and 19.3% [5]. In a study on 402 eyes of 257 patients with uveitis, Harbert et al. [13] found the rate of IOP elevation requiring treatment as 29.8% and secondary glaucoma rate as 9.6%. In a study on 161 eyes of 100 patients, Panek et al. [14] reported the secondary glaucoma incidence as 19.3%, whereas Elgin et al. [15] reported that it was 10.9% in patients with Behçet disease. In a recent study, secondary glaucoma occurred in more than 30% of patients with Vogt-Koyanagi-Harada disease [16]. In our study, secondary glaucoma was detected in 21.9% of the patients. Although the secondary glaucoma rate was close to those mentioned in the literature, it was still higher than the mean. The possible reason is that our clinic is a tertiary healthcare facility where patients with severe and chronic uveitis difficult to manage were referred. Another explanation for a higher incidence of secondary glaucoma is the fact that our study included a greater number of eyes with chronic uveitis in about two-thirds of cases when compared to eyes with acute uveitis. Of 67 eyes with secondary glaucoma, the most common uveitis localization was panuveitis in more than half of the cases that were followed by anterior uveitis in two-fifths of cases. This finding was similar to general uveitis localizations.

Secondary glaucoma due to chronic uveitis is more commonly seen than those in patients with acute uveitis. Indeed, IOP elevation after acute inflammation is generally transient and is responsive to anti-inflammatory drugs. Although humor aqueous production is reduced during the acute stage of uveitis, prostaglandins that were released during inflammation can cause IOP elevation from humor aqueous over-production. However, in eyes with chronic uveitis, recurrent inflammatory attacks, if untreated sufficiently with recent modern biological medications [17,18], can cause fibrosis, destruction, and fibrous membrane formation on the trabecular system with or without seclusio pupillae or rubeosis iritis. On the other hand, secondary glaucoma may develop due to topical, periocular, and systemic corticosteroid usage [19]. Herbert et al. [13] demonstrated that raised IOP was found in 26.0% of eyes with acute uveitis and 46.1% of eyes with chronic uveitis. Panek et al. [14] reported that secondary glaucoma was found in 12% of eyes with acute uveitis and 26% of the cases with chronic uveitis. In the present study, secondary glaucoma developed in 13.7% of cases with acute uveitis and 26.8% of the cases with chronic uveitis.

One of the most important and well-known ocular adverse effects of corticosteroids is the elevation of IOP, which varies depending on the substance and application route with some individual characteristics [20]. In uveitic eyes, raised IOP related to intraocular inflammation or corticosteroid use returns to normal shortly after the controlling of inflammation and withdrawal of corticosteroids [15]. Takahashi et al. [21] found secondary glaucoma in 18.3% of the patients and reported that 8.9% of these cases were caused by corticosteroids. In the present study, short-term elevation of IOP that was responsive to withdrawal of the drug was found in 3.5% of 307 eyes and subtenon or intravitreal corticosteroid injection caused IOP elevation in about 2.3% of cases, which was responsive to anti-glaucoma medications within two to 24 months with no visual field defects or glaucomatous optic nerve injury.

Medical and surgical treatment is more challenging in glaucoma secondary to uveitis than primary glaucoma. In our study, IOP was controlled by medical therapy in four-fifths of cases, and surgical treatment was performed in the remaining one-fifth of cases. Surgical requirement in secondary glaucoma was reported between 9% and 30% [13,14,22]. Nd:YAG laser iridotomy is one of the treatment options if a convex iris is detected with elevated IOP. Although iridotomy has a higher success rate in glaucoma cases with acute angle closure, it can still fail secondary uveitic glaucoma since iridotomy may be closed by the activation of inflammation, and failure rates have been reported to be 40-61% [23]. Sallam et al. [22] reported that eyes with secondary glaucoma had trabeculectomy with an anti-metabolite in 30 eyes, trabeculectomy without an anti-metabolite in three eyes, shunt implantation in six eyes, and iridectomy in one eye. In our study, Nd:YAG laser iridotomy was required in five patients. However, additional medication was needed in four patients to control IOP, whereas one patient required subsequent filtration surgery. Therefore, surgical iridectomy with filtration surgery may be performed in case of iridotomy closure [23].

Changes in conjunctival structures with an increased number of fibroblasts, lymphocytes, and macrophages due to used agents may increase the likelihood of failure in filtration surgeries for uveitic glaucoma, and uveitis attacks have to be suppressed for at least three months before filtration surgeries [24]. The success rate varies from 51% to 90% in trabeculectomy with anti-metabolites [24,25]. Ceballos et al. [25] reported success rates in glaucoma secondary to uveitis as 78% in the first year and 62% in the second year. Elgin et al. [15] reported the success rate of trabeculectomy with MMC as 82.6% in the first year [5]. Yalvaç et al. reported the success rates of trabeculectomy with MMC performed in eyes with glaucoma secondary to Behçet disease as 83.3%, 76.2%, 70.0%, 66.7%, and 62.5% at the year one, two, three, four, and five, respectively [26]. In our study, trabeculectomy with MMC was performed in seven eyes with a follow-up period ranging from three months to six years. In one patient who had been followed in our clinic, trabeculectomy was performed in another facility. IOP was controlled without drugs in about three-fourths of cases and with anti-glaucomatous agents in only two (28.5%) of the patients who underwent trabeculectomy with MMC. One patient used the ß-blocker+CAI combination, while the other patient used the ß-blocker+CAI combination and sympathomimetic. Hypotonia developed after surgery in one patient who underwent bleb revision and subconjunctival autologous blood injection. In another patient, IOP remained higher beginning from the first day after surgery. Medical treatment was added, and bleb revision was performed. A second trabeculectomy with MMC was performed eight months after the first operation as the IOP remained uncontrolled. These patients had end-stage Behçet disease. There was chronic panuveitis and uveitis attacks that could hardly be controlled. Taken together, our success rate was 71.4% for trabeculectomy with MMC in patients with glaucoma secondary to uveitis. However, smaller sample sizes in the surgical group and limited follow-up periods after surgery make it difficult to compare our success rate.

Express mini glaucoma shunt is an alternative aqueous drainage implant with high stability in the anterior chamber [27,28]. The effect of the express mini shunt is similar to standard trabeculectomy, but fewer complications have been reported when compared to standard trabeculectomy and other aqueous drainage shunts. Fewer postoperative reactions and bleb fibrosis are encountered without iridectomy. In our study, express mini shunt was performed in one patient, and IOP regulation was achieved without drug usage during the 12-month follow-up period.

There are three limitations to the study. First, the sample size in the surgical group as a result of secondary glaucoma was small. Second, the follow-up periods after surgery were short, making it difficult to compare our success rate with previous reports. Third, our study lacks a comprehensive evaluation of surgical necessity in children as the number of children was small.

## Conclusions

Uveitis is an ocular disease that can be encountered in association with several systemic and ocular diseases. The etiology is unknown in some cases, and such patients should be monitored closely. Uveitis can be encountered in all ages and sexes and may result in severe complications. Indeed, secondary glaucoma is one of the most important complications of uveitis, which may result in loss of vision. Although IOP is normal or decreased in some forms of uveitis, the risk of glaucoma should be kept in mind in all cases with uveitis, and regular measurements of IOP should be performed. Early diagnosis and appropriate treatment could prevent potential complications.
